# Auditory Neural Activity in Congenitally Deaf Mice Induced by Infrared Neural Stimulation

**DOI:** 10.1038/s41598-017-18814-9

**Published:** 2018-01-10

**Authors:** Xiaodong Tan, Israt Jahan, Yingyue Xu, Stuart Stock, Changyow Claire Kwan, Carmen Soriano, Xianghui Xiao, Jaime García-Añoveros, Bernd Fritzsch, Claus-Peter Richter

**Affiliations:** 1 Department of Otolaryngology, Northwestern University Feinberg School of Medicine, 320 E. Chicago Avenue, Searle 12-561, Chicago, IL 60611 USA; 2 Department of Biology, University of Iowa, 129 E. Jefferson Street, Iowa City, IA 52242 USA; 3 Department of Cell and Molecular Biology, Northwestern University Feinberg School of Medicine, 303 E. Chicago Avenue, Chicago, IL 60611 USA; 4 Advanced Photon Source, Argonne National Laboratory, 9700 South Cass Avenue, Argonne, IL 60439 USA; 5 Departments of Anesthesiology, Physiology, and Neurology, Northwestern University Institute for Neuroscience, Ward 10-070, 303 East Chicago Avenue, Chicago, IL 60611 USA; 6 Department of Biomedical Engineering, Northwestern University, 2145 Sheridan Road, Tech E310, Evanston, IL 60208 USA; 7 The Hugh Knowles Center, Department of Communication Sciences and Disorders, Northwestern University, Frances Searle Building, 2240 Campus Drive, Evanston, IL 60208 USA

## Abstract

To determine whether responses during infrared neural stimulation (INS) result from the direct interaction with spiral ganglion neurons (SGNs), we tested three genetically modified deaf mouse models: *Atoh1-cre; Atoh1* ^*f/f*^ (*Atoh1* conditional knockout, CKO), *Atoh1-cre; Atoh1* ^*f/kiNeurog1*^ (*Neurog1* knockin, KI), and the *Vglut3* knockout (*Vglut3* ^−/−^) mice. All animals were exposed to tone bursts and clicks up to 107 dB (re 20 µPa) and to INS, delivered with a 200 µm optical fiber. The wavelength (λ) was 1860 nm, the radiant energy (Q) 0-800 µJ/pulse, and the pulse width (PW) 100–500 µs. No auditory responses to acoustic stimuli could be evoked in any of these animals. INS could not evoke auditory brainstem responses in *Atoh1* CKO mice but could in *Neurog1* KI and *Vglut3* ^−/−^ mice. X-ray micro-computed tomography of the cochleae showed that responses correlated with the presence of SGNs and hair cells. Results in *Neurog1* KI mice do not support a mechanical stimulation through the vibration of the basilar membrane, but cannot rule out the direct activation of the inner hair cells. Results in *Vglut3* ^−/−^ mice, which have no synaptic transmission between inner hair cells and SGNs, suggested that hair cells are not required.

## Introduction

Neural stimulation with photons has been proposed for a next generation of cochlear implants (CIs). The potential benefit of photonic stimulation is its spatially selective activation of small neuron populations. Stimulating smaller spiral ganglion neuron (SGN) populations along the cochlea provides a larger number of independent channels to encode acoustic information. Hearing could be restored at a higher fidelity (for a review see Richter and Tan^1^) and performance in noisy listening environments as well as music appreciation are likely to improve.

Two methods for optical stimulation have been proposed, optogenetics and infrared neural stimulation (INS)^1–4^. Optogenetics requires a viral vector to express photosensitive ion channels in the membrane of the target neurons^3,5^, the SGNs in Rosenthal’s canal. INS does not require such treatment because during INS, the fluid in the target tissue absorbs the photons and the energy is converted into heat^6–10^. The resulting rapid temperature change (dT/dt) leads to capacitive changes of the cell membrane^8,11–13^, activation of temperature sensitive ion channels, such as transient receptor potential channels^9,14,15^, changes in gating dynamics of potassium and sodium channels^12^, modulations of GABAergic transmission^16^, activation of a second messenger^17^, calcium release in the cell^7,8,18,19^, or to mechanical events such as stress relaxation waves with measurable pressure^10,17,20–22^. While several laboratories have been able to evoke auditory response using INS, controversies exist regarding whether the stimulation of the auditory system is dominated by laser-induced photoacoustic effects^23–25^ or by direct stimulation of spiral ganglion neurons (SGNs)^26^.

With the intention to implement neural stimulation with infrared light into neural prostheses, such as cochlear implants, it is important to determine whether stimulation results from the direct interaction of the radiation with spiral ganglion neurons or whether functional hair cells are required because many cochlear implant users lack most of their hair cells. To answer this question, an animal model that has no hair cells but has functional spiral ganglion neurons is needed. Unfortunately, it is not possible to eliminate all the auditory hair cells in normal hearing animals without affecting SGN viability due to required neurotrophic support during development^27^. Residual hearing was often observed in deafened animals, especially at low frequencies and at high sound levels^28^. Even though the pressure in the cochlea generated by laser pulses was measured and found unlikely to induce an acoustic response in deaf animals^10^, this does not constitute direct evidence against a photoacoustic effect involving hair cells^23,25^. The factors causing hair cell loss also effect the function and the survival of SGNs, both in acute treatments and chronic animal models^29,30^, as well as in humans^31^. The inability to evoke compound action potentials (CAPs) from the auditory nerve or to evoke auditory brainstem responses (ABRs) using laser stimulation in some deaf animal models might simply be due to fewer SGNs available in long-term deaf animals or to the damage of neurons after acute deafening.

The objective of this study is not to determine the mechanism of INS but to pinpoint the target structure(s) for INS in cochleae of deaf animals. The experiments were designed to rule out that the responses in deaf animals result from (1) a pressure wave generated by the irradiation with infrared light, which vibrates the basilar membrane and stimulates hair cells; or (2) transmitter release caused by the direct irradiation of hair cells. Three models of deaf mice were used. (1) A “self-terminating” *Atoh1* conditional knockout (*Atoh1-cre; Atoh1* ^*f/f*^, referred to as *Atoh1* CKO) mouse model which suffers from a complete inner hair cell (IHC) loss and a massive outer hair cell (OHC) loss^32^. (2) A *Neurog1* knockin (*Atoh1-cre; Aoth1* ^*f/kiNeurog1*^, referred to as *Neurog1* KI) mouse where *Neurog1* substitutes *Atoh1* function and rescues a large number of hair cells. IHCs and OHCs are more developed but their stereocilia are partially undifferentiated. Despite hair cell rescue, no hearing restoration is achieved^33^. (3) A vesicular glutamate transporter-3 knockout (*Vglut3* ^−/−^) mouse model which has normal hair cell counts and functional SGNs. However, no neurotransmitter release occurs in these mice, even when hair cells are directly depolarized by electrical current or high extracellular potassium concentrations^34–36^. The absence of glutamate release in *Vglut3* ^−/−^ mice is due to a loss of vesicle filling. Consequently, INS could not trigger neurotransmitter release in these synapses.

Typically, SGNs are stimulated through mechanical vibration of the basilar membrane, which leads to a stimulation of hair cells followed by release of neurotransmitter. In *Atoh1* CKO and the *Neurog1* KI mice, normal stimulation of neurons is disrupted due to the disorganized cochlear structure, and acoustic stimuli do not result in auditory responses. If INS induces a response in the auditory system of these animals, it most likely does not come from a pressure wave vibrating the basilar membrane but from a direct interaction with either the neurons or the hair cells. For the two mouse models, a direct interaction of the radiation with the hair cells is possible, as has been reported before for the vestibular hair cells *in vivo* ^37–39^ and *in vitro* studies^40,41^. Irradiation of the hair cells could trigger transmitter releases and stimulate SGNs. To distinguish between stimulation of hair cells and direct stimulation of SGNs, a third deaf mouse model (*Vglut3* ^−/−^) was employed. As described, the glutamate transporter is affected and no transmitter can be released from the inner hair cells to stimulate SGNs. Auditory responses evoked by irradiation of cochleae of *Vglut3* ^−/−^ mice would indicate that SGNs are directly stimulated.

## Results

Our results showed that all animals of the three mouse models are severely deaf. While some of the animals have considerable counts of surviving SGNs, others have significant loss or almost no remaining neurons as determined from histology. INS was applied to 3–5 month old animals of the three strains of transgenic mice to test whether auditory neural responses could be induced in the animal models. The comparison between *Atoh1* CKO and *Neurog1* KI demonstrated that INS is unlikely related to the mechanical stimulation of the basilar membrane and subsequent mechanoelectrical transduction by hair cells. Measurements in *Vglut3* ^−/−^ mice demonstrate that INS evoked auditory responses despite the inability of hair cells in these mice to release glutamate transmitter. Taken together, the data show that INS can directly stimulate SGNs.

### None of the Three Transgenic Mouse Strains Showed ABRs to Acoustic Stimulation

**Figure 1:**
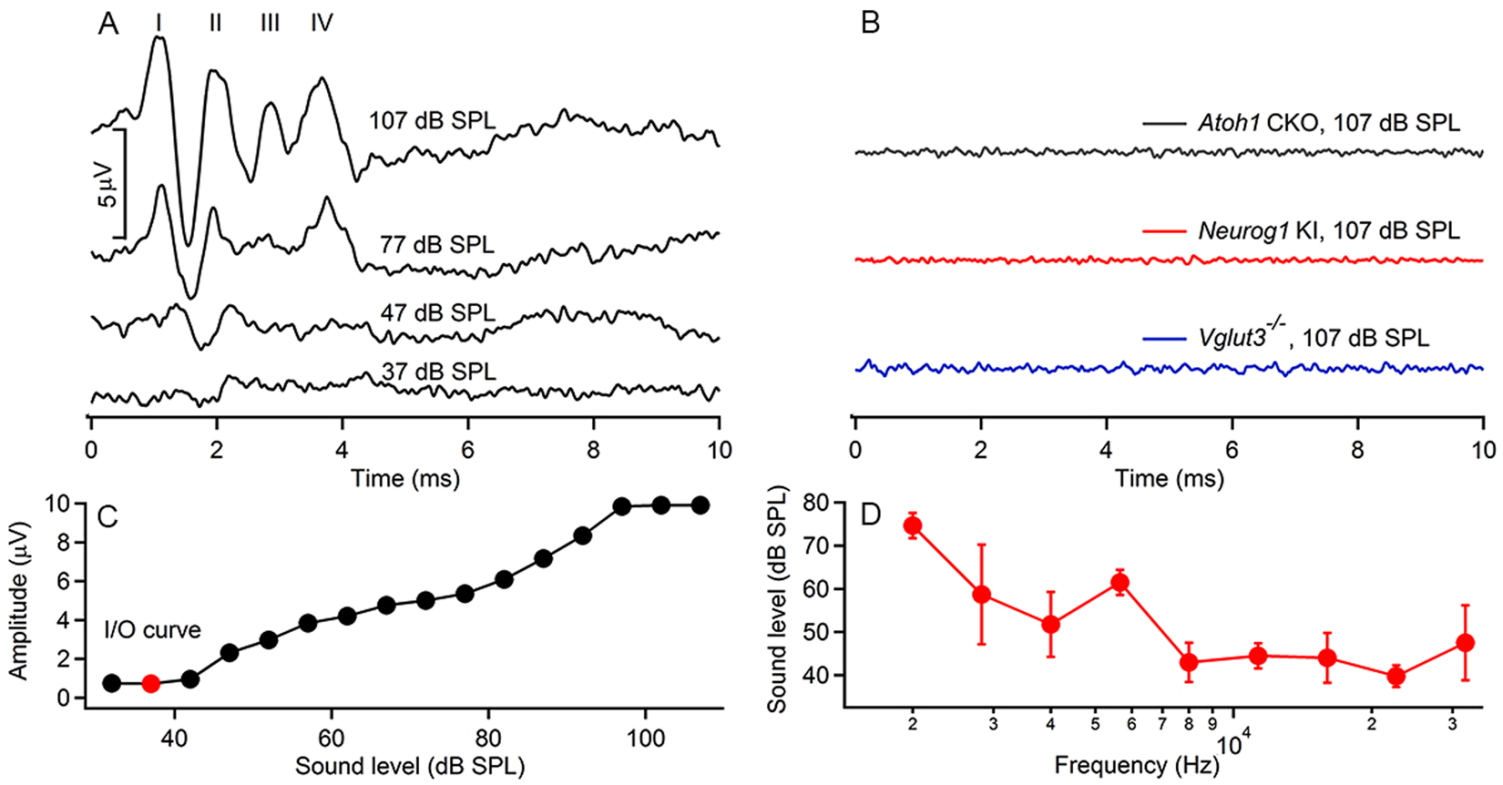
Acoustically (clicks) evoked ABRs (aABRs) could be recorded in normal hearing mice **(A)** but not in the three strains of congenitally deaf mice **(B)**. Multiple peaks can be identified in the aABR waveforms of normal hearing animals (marked as I-IV in panel A), which gradually disappeared with the decrease in sound level. No aABR was induced in the three strains of congenitally deaf animals. The sound stimuli used in (**A**) and (**B**) were clicks, and similar results were obtained with tonal stimulations. The input/output (I/O) curve in response to clicks in a normal hearing animal is plotted in (**C**), with a threshold at 37 dB SPL (red dot). The threshold at each frequency of all the normal hearing animals used in this study was plotted in (**D**).

Cochlear function was assessed by recording auditory brainstem responses (aABRs) to pure tone stimuli and to acoustic clicks. Figure 1 shows the results of the aABR measurements to acoustic clicks. Typical aABRs from normal hearing animals are shown in Fig. 1A. At high sound levels multiple maxima can be identified (Fig. 1A, the trace marked “107 dB SPL”). Each maximum originates from the neural activity at different sites along the auditory pathway, from the auditory nerve to the midbrain^42,43^. As the sound level was lowered, the maxima decreased in amplitude until they were no longer distinguishable from the background noise of the recording. Figure 1C shows the responses at different sound levels for a normal hearing animal in response to clicks. The hearing threshold was 37 dB SPL (sound pressure level re 20 µPa), shown by the red dot. In all 6 normal hearing control animals an aABR was recorded and was on average 10.5 ± 2.4 µV. The average threshold was 35.4 ± 3.5 dB SPL for clicks. The thresholds for tones at different frequencies were also measured and are shown in Fig. 1D.

In contrast to normal hearing animals, acoustic stimuli failed to evoke an aABR in all three strains of genetically manipulated mice (Fig. 1B). *Atoh1* CKO (gray trace), *Neurog1* KI *(*red trace) and *Vglut3* ^−/−^ (blue trace). None of the three strains of mice had any residual hearing as determined by stimulating with acoustic clicks or pure tones, even at the highest possible sound levels (107 dB SPL). This data confirmed previous findings^32,33,36^.

### Distortion product otoacoustic emissions (DPOAEs) were recorded in Neurog1 KI and Vglut3^−/−^ Mice but not Atoh1 CKO Mice

**Figure 2:**
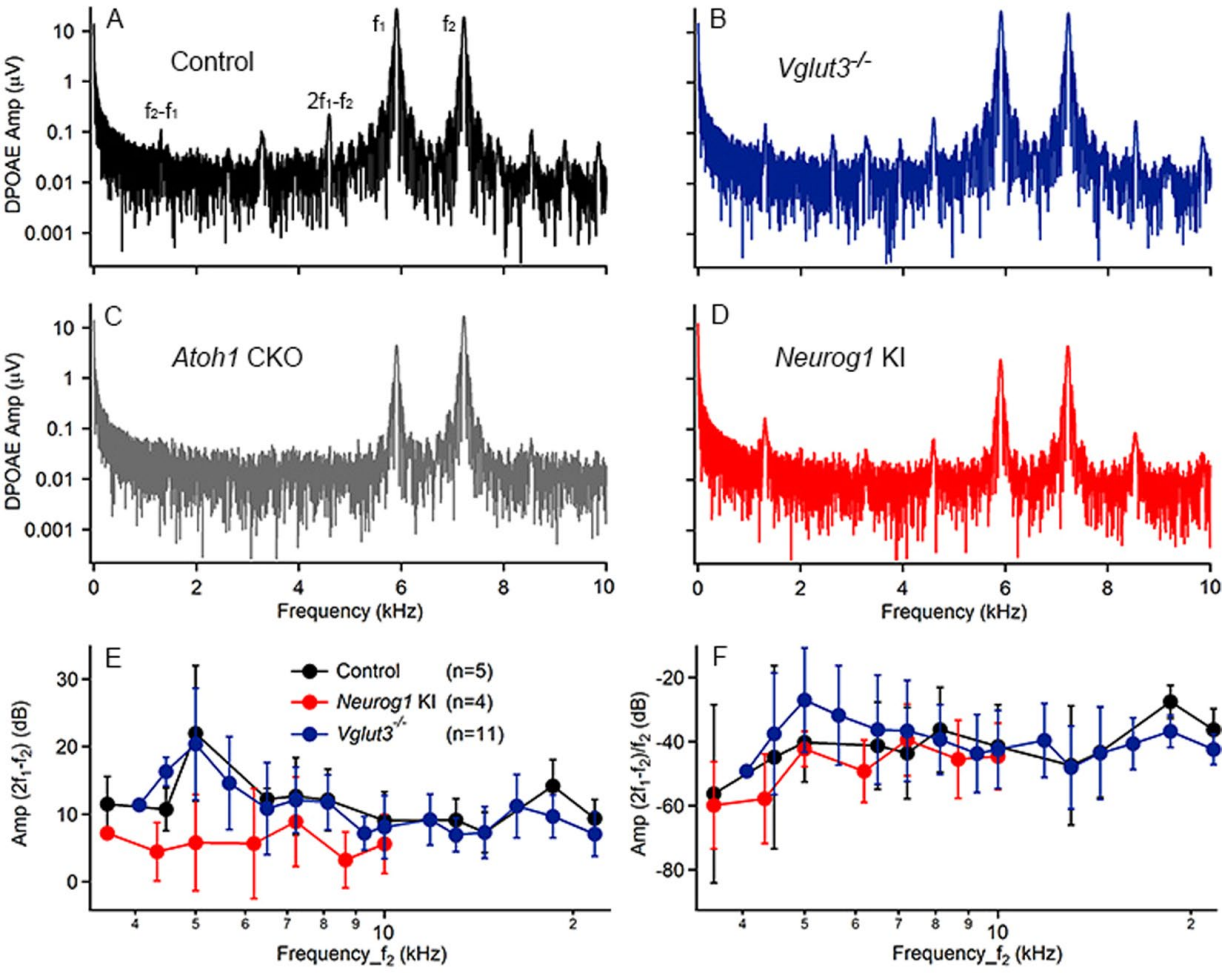
DPOAE measurements in different mouse strains. DPOAEs were measured in Control **(A)**, *Vglut3* ^−/−^ **(B)** and *Neurog1* KI **(D)** but not in *Atoh1* CKO **(C)** mice. DPOAE waveforms induced by the same stimuli (f_1_ = 5.9 kHz and f_2_ = 7.2 kHz, both at about 60 dB SPL) from the 4 different animals were used as examples. The stimuli (f_1_ and f_2_ as well as the cubic (2f_1_ -f_2_) and quadratic (f_2_ -f_1_) distortion products were marked in **(A)** and can be extended to other panels. Note that no peak of DPOAE was measured in *Atoh1* CKO mice **(C)**. The cubic distortion product (2f_1_-f_2_) **(E)** and its ratio to f_2_ **(F)** were also plotted against cubic f_2_. The noise level was at about 0 dB for E.

DPOAEs constitute a measure for the nonlinearity of the cochlea, which is assumed to originate from an active process driven by the OHCs^43,44^. To assess the function of the OHCs in the three strains of transgenic mice, DPOAEs were measured and shown in Fig. 2. Robust DPOAEs were evoked in most normal hearing animals (5 out of 6) over the entire range of the frequencies used for stimulation, with an f_2_ ranging from 3.5 to 21.6 kHz. A typical spectrum of the DPOAEs generated with f_1_ = 5.9 kHz and f_2_ = 7.2 kHz is shown in Fig. 2A. The 2 stimulus frequencies are indicated by the 2 peaks marked f_1_ and f_2_. A cubic distortion product (2f_1_-f_2_) was clearly seen as well as the quadratic distortion product (f_2_-f_1_), which was at a lower frequency range. Although none of the three transgenic strains had cochlear responses at any frequency, robust DPOAEs were observed in *Vglut3* ^−/−^ (11 out of 12) and *Neurog1* KI (4 out of 6) mice but not in *Atoh1* CKO mice (0 out of 6) (Fig. 2B–D). This absence of DPOAE in *Atoh1* CKO is consistent with the reported massive loss of OHCs by 6 weeks of age^32^. To gain a comprehensive view on the nonlinearity in the cochlea, including the function of the OHCs, the cubic distortion products (2f_1_-f_2_) measured at different f_1_/f_2_ pairs were plotted against f_2_ in Fig. 2E. The DPOAEs induced in *Vglut3* ^−/−^ mice were similar to those of controls in both amplitude and frequency range. However, the DPOAEs induced in *Neurog1* KI mice were notably smaller, possibly reflecting variable OHC disorganizations^33^. To account for variation in the measurements, the data were normalized by calculating the difference between 2f_1_-f_2_ and f_2_ and plotting the resulting values against f_2_ (Fig. 2F). These results indicated functional OHCs in the cochleae of the *Neurog1* KI and *Vglut3* ^−/−^ mice.

### Optical Responses were evoked in Neurog1 KI Mice but not Atoh1 CKO Mice

**Figure 3:**
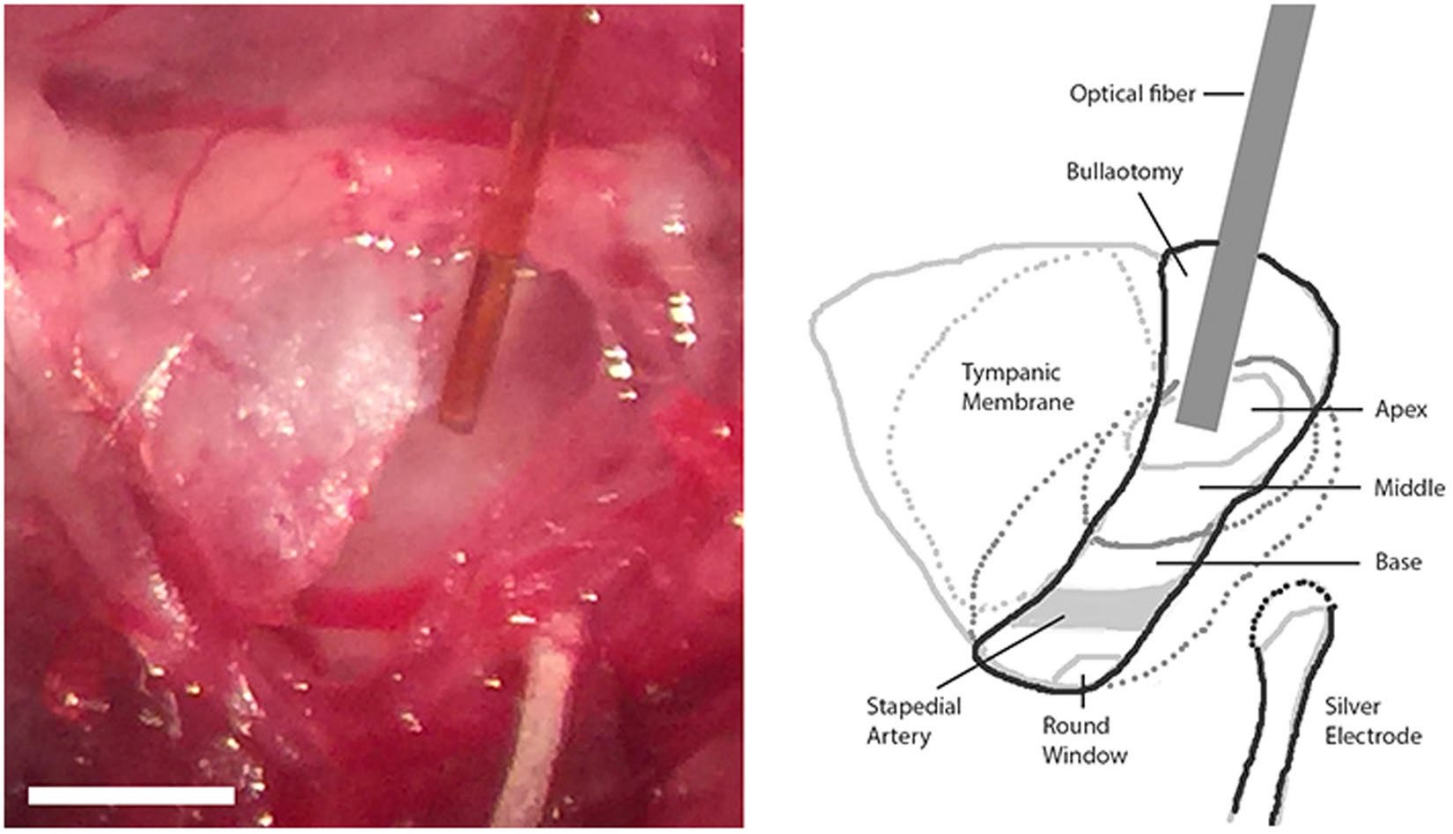
The image (left) and the sketch (right) show the optical stimulation and the recording sites. A 2.0 × 0.6 mm^2^ bullotomy was made, through which the 3 turns of the cochlea were visible and accessible. An optical fiber was placed on the apical cochlea wall for INS. A silver wire was placed outside the bulla for ABR recording. The structures shown by dashed lines were only estimations and might not be precise. Scale bar: 1 mm.

Optical pulses (wavelength (λ) = 1860 nm, radiant energy (Q) = 0–800 µJ/pulse, radiant exposure (RE) = 0–25.5 mJ/cm^2^, and pulse width (PW) = 100–500 µs) were delivered through a low H_2_O optical fiber with a core diameter of 200 µm. The configuration of the oABR measurement is shown in Fig. 3. The resulting temperature change has not been measured directly because the modiolus is not accessible in the closed cochlea but has been estimated as describe previously^45^. During INS the instantaneous temperature rise after a laser pulse (t = 0) was calculated with the assumption that water is the dominant absorber of photons using the following equation:

$$T(z,0)=\frac{{\mu }_{a}H(z)}{\rho c}$$

where µ_a_ is the wavelength-dependent absorption coefficient of the material, which would be 14.2 cm^−1^ at λ = 1860 nm for water; *H(z)* is the radiant exposure at z along the optical axis; *ρ* is the density of water, which is about 1000 kg/m^3^; and the corresponding specific heat c of the tissue is 4200 J/kg °C. The calculated temperature rise for the given parameter is 8.7 °C at the tip of the optical fiber. For the maximum radiant energy used in the present experiments, the temperature rise would be calculated to be 2.9 °C at the modiolus.

**Figure 4:**
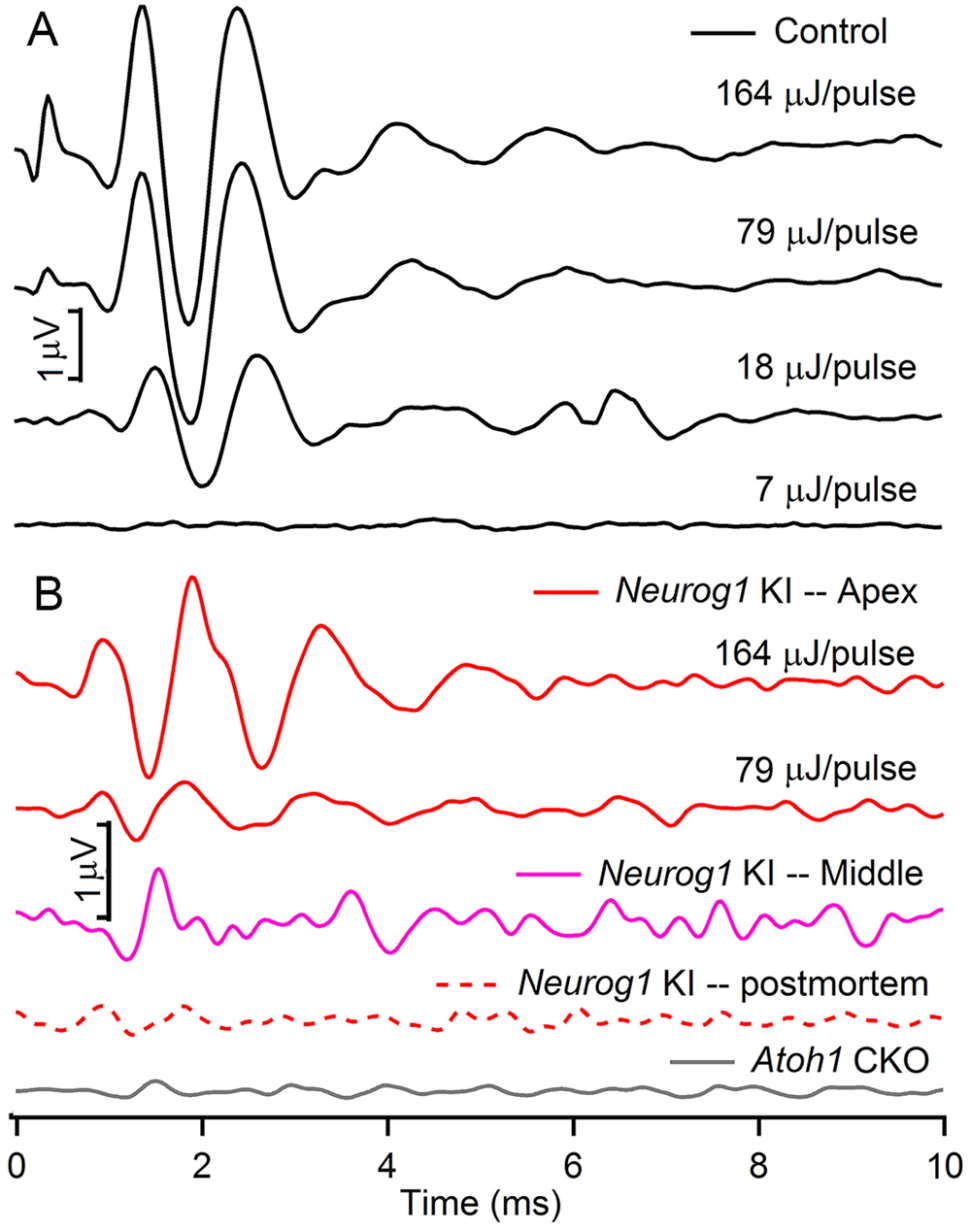
Example waveforms of the optical ABRs (oABRs) induced in normal hearing **(A)**, *Neurog1* KI and *Atoh1* CKO mice **(B)**. The pusle length of the optical stimulation was 100 µs delivered with a repetition rate of 5 Hz. The oABRs also showed multiple peaks like the aABRs and the magnitude decreased with the decrease of the energy level. oABRs induced in *Neurog1* KI mice showed spatial differences. The maximal response was induced at the apex (red traces) while the magnitude decreased at the middle (pink trace, the energy level = 164 µJ/pulse) and disappeared at the base (trace not shown) and postmortem (red dashed trace). No oABR was induced at any location of the *Atoh1* CKO mice (gray trace, the energy level = 164 µJ/pulse).

Representative waveforms for optical evoked ABRs (oABRs), recorded from control group animals and the three transgenic mouse strains are shown in Fig. 4. In normal hearing animals, oABRs showed similar waveforms as aABRs. Multiple peaks representing neural activity from different locations along the auditory pathway can be seen (Fig. 4A). However, the maximal amplitude of the oABRs is smaller than that of the aABRs. The largest average oABR amplitude from 4 normal hearing animals was 3.9 ± 1.0 µV (mean ± SD), which is significantly less than the 9.4 ± 2.1 µV amplitude of the aABR measured in animals with normal cochlear function to acoustic click stimuli. The laser parameters were: λ = 1860 nm, Q = 0–164 µJ/pulse, and PW = 100 µs. The threshold radiant energy to evoke an oABR was 12.3 ± 4.6 µJ/pulse, similar to what we have previously measured in guinea pigs^26^.

**Figure 5:**
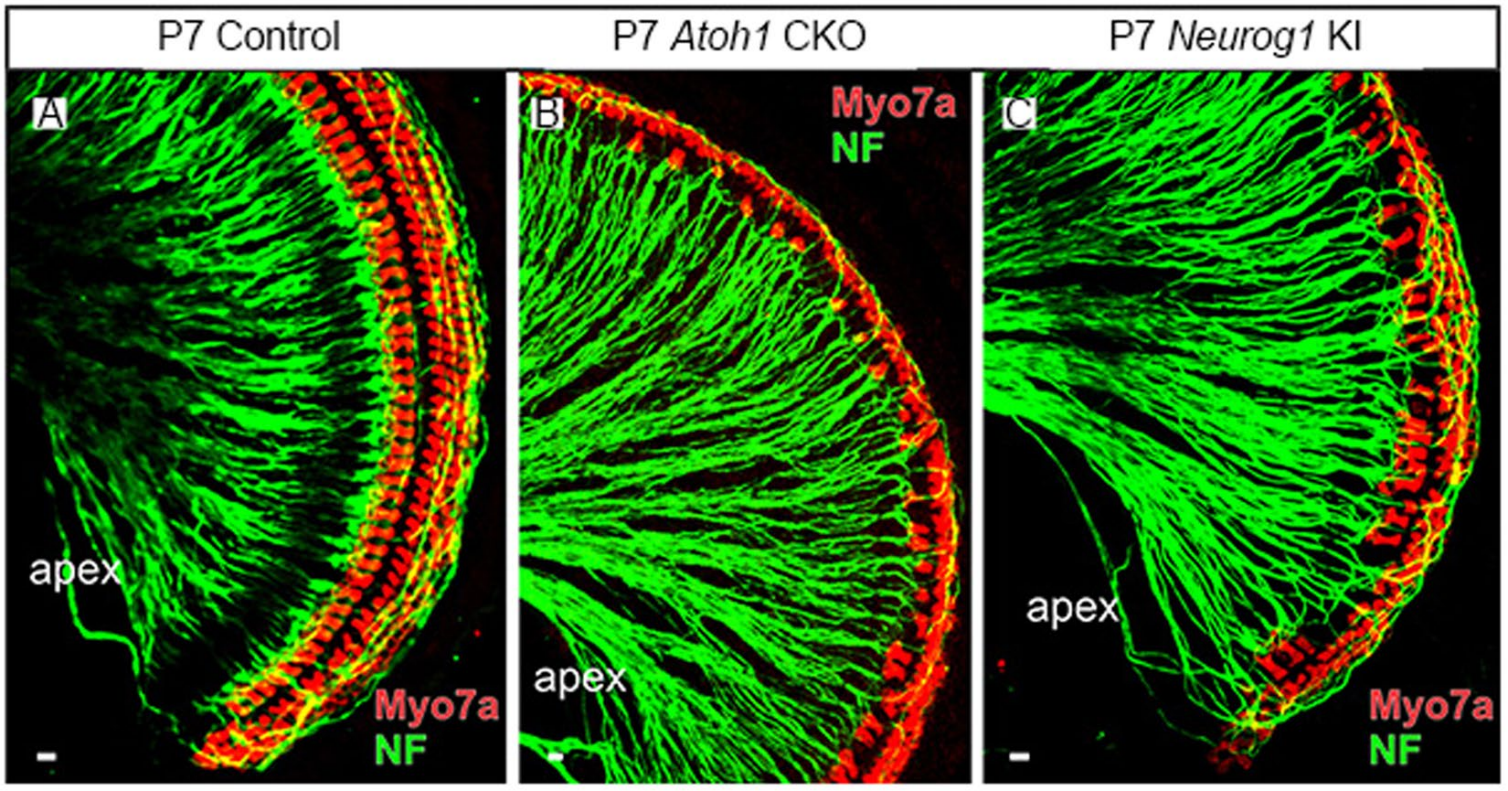
Immuno-staining of cochlear whole mounts showing the morphology of auditory nerve fibers in Control, *Atoh1* CKO and *Neurog1* KI animals at P7. Note that the nerve fibers appear intact in both *Atoh1* CKO and *Neurog1* KI mice at P7. NF, Neurofilament. Scale bars at lower left corners indicate 10 µm.

As reported previously, *Atoh1* CKO mice have only a few remaining IHCs at the cochlear apex and only one row of OHCs^32^ while *Neurog1* KI mice have most of the hair cells rescued^33^. Both strains had intact auditory nerve fibers postnatally, as shown with the Neurofilament and Myo7a immunochemistry (Fig. 5), but were completely deaf. No responses to acoustic stimuli could be recorded.

The results have shown that, oABRs could be induced in 4 out of 6 *Neurog1* KI mice when the optical fiber was placed at the apical to middle turns, but not at the basal turn, near the round window of the cochlea (Fig. 4B). The response was largest at the apex, with a maximum of 1.8 ± 0.6 µV (n = 4, Fig. 4B, the red traces), while a smaller or no response was recorded while the fiber tip was placed at the middle turn (1.0 ± 0.5 µV, n = 3, Fig. 4B, the pink trace). All responses disappeared postmortem (Fig. 4B, the red dashed trace). On the contrary, no response was evoked in *Atoh1* CKO mice at any locations along the cochlea (0 out of 6, the gray trace). Those animals had very few SGNs, which could be targeted by the infrared light (see also below).

### oABRs were evoked in Vglut3^−/−^ Mice

**Figure 6:**
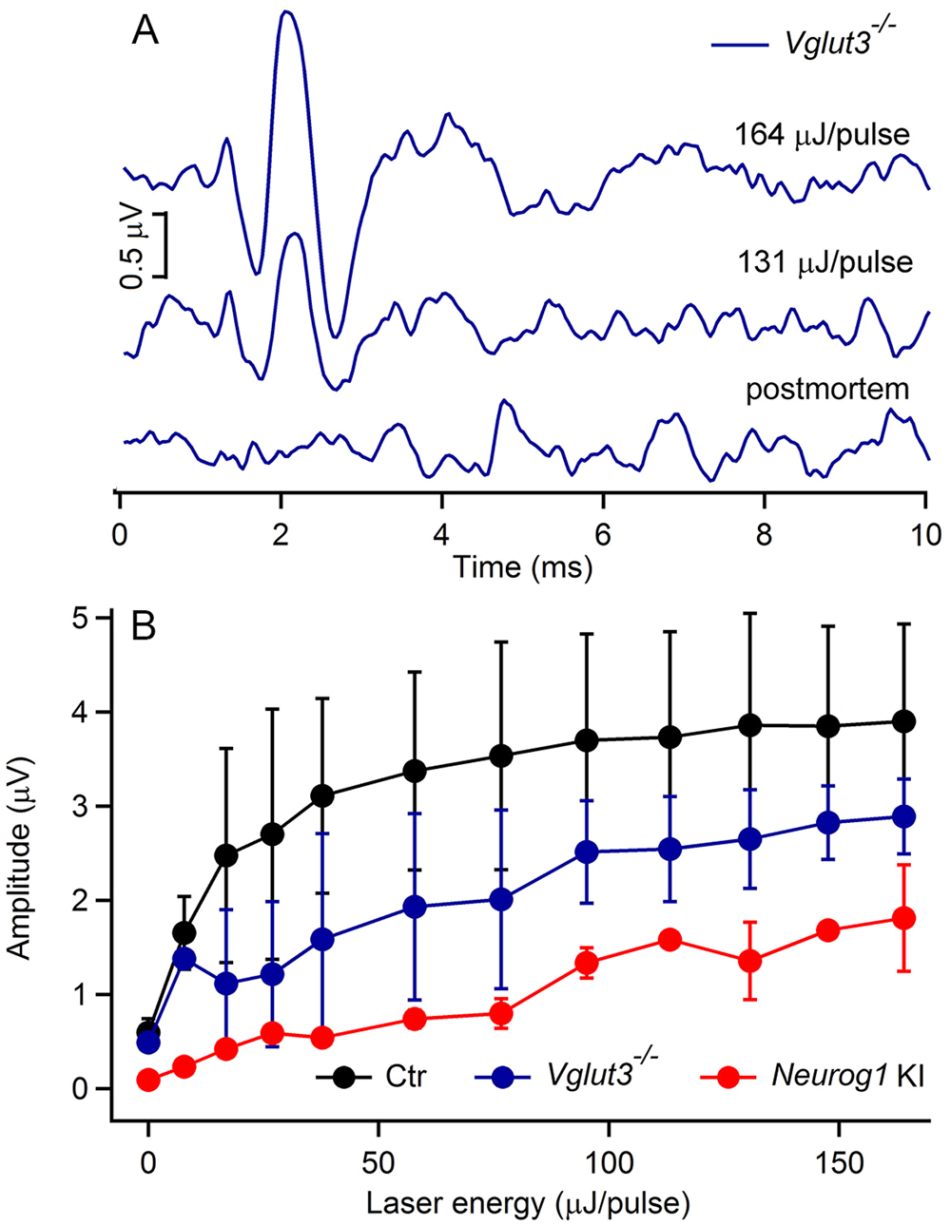
The oABRs induced in *Vglut3* ^−/−^ mice **(A)** and the input/output curves of the oABRs induced in control, *Vglut3* ^−/−^ and *Neurog1* KI animals **(B)**. Ctr: control animals.

The results obtained from *Atoh1* CKO and *Neurog1* KI mice suggest that hair cells are required for INS. Since the hair cells are not likely stimulated through the vibration of the basilar membrane and subsequent deflection of their stereocilia bundles as occurs in a pristine cochlea, transmitter release following a direct irradiation of the cells must occur. To further determine the involvement of hair cells in INS, the same infrared pulses as used in the previous experiments were delivered to *Vglut3* ^−/−^. No responses to INS should be seen in *Vglut3* ^−/−^ mice if the target for the stimulation are the hair cells, because the synaptic connection between the IHC and SGNs is broken. Nevertheless, robust oABRs were evoked in *Vglut3* ^−/−^ mice. As shown in Fig. 6, oABRs were evoked at various locations along the cochlea from the apex to the base (5 out of 6). The maximal amplitude was 2.9 ± 0.4 µV (n = 3). For 2 animals a longer PW (500–1000 µs) was used to evoke a response. The wavelength was used to determine whether a response could be evoked in those animals. However, because the laser parameters used for stimulation differed from those used for the rest of the animals, the results were excluded from the statistics and the plot.

For the animals included in the statistics, the threshold for INS was 17.8 ± 9.4 µJ/pulse, (n = 3). The threshold radiant energy for an oABR in *Vglut3* ^−/−^ mice was comparable to that evoked in normal hearing animals. The threshold of 2 animals was not determined. The input/output functions of evoked oABRs of control (black), *Vglut3* ^−/−^ (blue) and *Neurog1* KI (red) animals are shown in Fig. 6B. The oABRs evoked in *Vglut3* ^−/−^ mice were larger than those of *Neurog1* KI mice but smaller than those of the controls.

### Synchrotron X-ray microCT Imaging Revealed the Requirement of SGNs for INS

**Figure 7:**
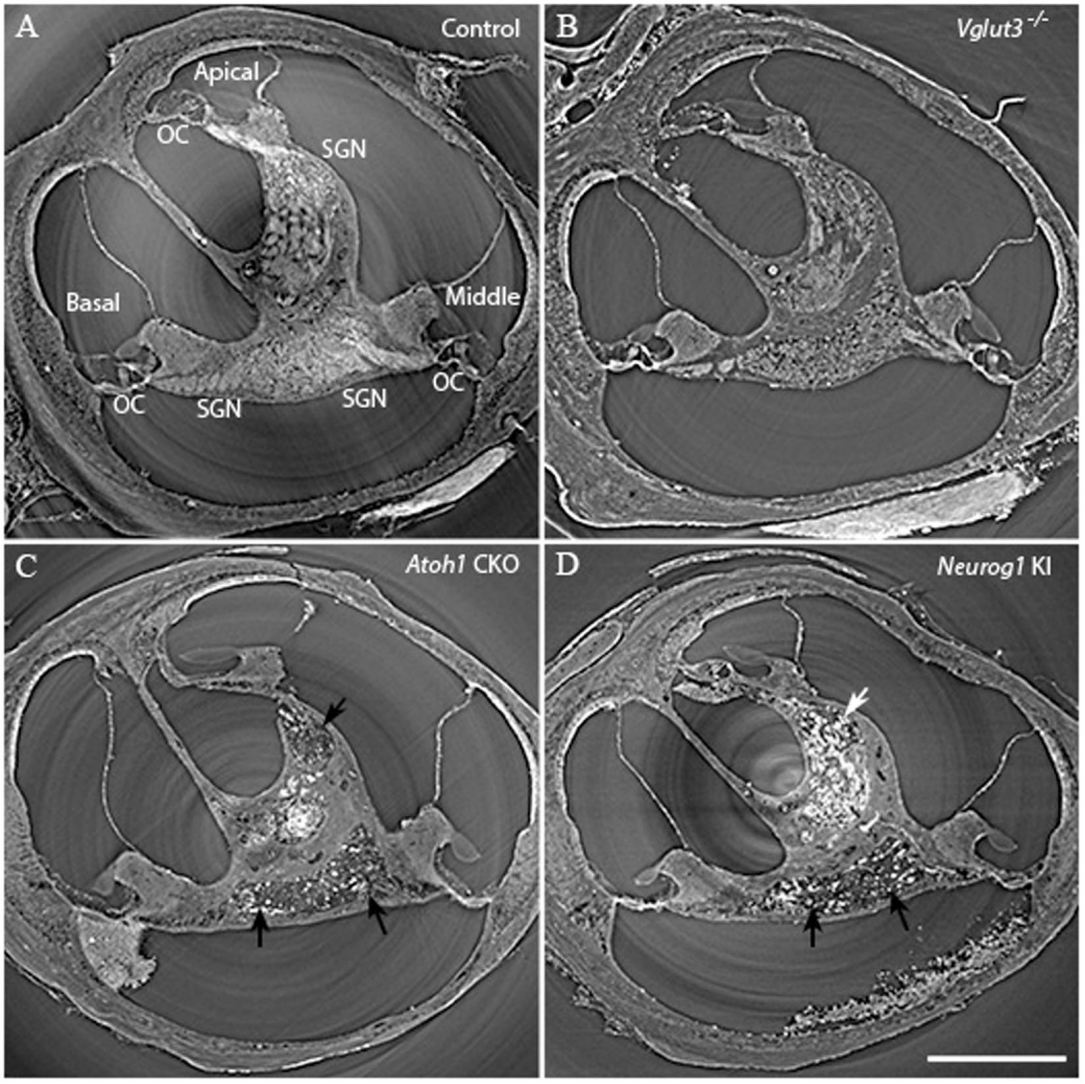
Synchrotron X-ray micro-computed tomography images of the cochleae of the 4 mice strains (at the age of 3–5 months). All the structures of the cochlea were intact in control **(A)** and *Vglut3* ^−/−^ **(B)** mice. However, the entire organ of Corti (OC) was lost in the *Atoh1* CKO mice **(C)**. SGNs were also mostly lost in all the three cochlear turns as indicated by the black arrows. **(D)** In *Neurog1* KI mice, the OC and SGNs survived at the apical (white arrow) but were reduced in the middle to basal cochlea (black arrows). Scale bar in panel D (for all panels): 0.5 mm.

To visualize the morphological status of each cochlea, cochleae were harvested for imaging at the end of the experiments. The samples were prepared as described for imaging at the Advanced Photon Source (APS)^46^ at Argonne National Laboratories. After reconstruction, the 3D stack of the images can be virtually sliced at any angle. Figure 7 shows a virtual section of the 3D set of reconstructed microCT images, from the control and the three strains of transgenic animals. The section crosses through the modiolus roughly at the middle turn, at an angle of about 45 degrees. Three turns of the cochlea are visible in this image slice. The images from control (Fig. 7A) and *Vglut3* ^−/−^ (Fig. 7B) animals show presence of hair cells and SGNs at age 3–5 months. The nerve fibers in the modiolus connecting hair cells and the neurons in the brainstem can be seen in all the 3 turns of the cochleae. However, the bodies of the SGNs of the *Vglut3* ^−/−^ mice were smaller and more granular than those of the controls, just like the documented changes of SGNs in deaf animals^47–49^. On the other hand, no hair cells were seen at any location along the cochlea of *Atoh1* CKO mice. These cochleae have a flat basilar membrane with the entire organ of Corti (OC) missing. In these samples, nerve fibers and the SGNs were also lost, with only sporadic neuronal bodies left in the Rosenthal’s canal (Fig. 7C). For *Neurog1* KI mice, hair cells and intact structure of the OC were observed at the apical but not the middle or basal parts of the cochlea. In accordance, SGNs are present at the apical part but were almost completely lost from the middle to basal parts (Fig. 7D). These results are highly consistent with the electrophysiological recordings, in that the neural response induced by the optical stimulation was spatially correlated with the presence of SGNs.

## Discussion

The results of the study support the view that the responses in deaf animals to INS (1) are not dominated by a pressure wave generated by the irradiation with infrared light vibrating the basilar membrane and stimulating hair cells, (2) are not the result of transmitter release caused by the direct irradiation of hair cells, and (3) are therefore due to the direct activation of SGNs. The spatially and temporally confined heating from INS leads temperature changes and action potential generation by one or more of the described and published mechanisms (see introduction and discussion below).

### The controversy: is direct neural stimulation of SGNs through irradiation with infrared light possible?

Nerve stimulation with mechanical stimuli, including pressure waves has been reported before^50,51^. This method of stimulation has been considered a possible mechanism for INS of the sciatic nerve. To test this view, optical coherence tomography was used to measure displacements of the nerve’s surface, which increased with increasing radiant energy. Near stimulation threshold the displacements were about 300 nm^6^. To further explore the question whether a mechanical event leads to the stimulation of the nerves during INS, an indenter, which modeled the pressure profile of the laser pulse was used to mechanically probe the nerve. No compound nerve action potential was evoked^6^. It has been concluded from the experiments that a mechanical stimulus does not play a significant role during INS of peripheral nerves. However, such pressure waves are important when the cochlea is functional. Teudt *et al*. provided the first experimental evidence that a laser induced pressure wave during INS can evoke a response in an animal with a normal functioning cochlea^20^. In subsequent experiments, this finding was solidified by demonstrating that irradiation with infrared light results in an audible pressure wave^10,21,22^. Ren *et al*.^52^ induced a photomechanical movement of the basilar membrane through irradiation of the basilar membrane with a 930 nm laser. The response was a local oscillatory vibration of the basilar membrane, which was interpreted as the results by the cochlear amplification. The study by Ren *et al*.^52^ provides the most direct evidence of the activation of OHCs with a pulsed laser^52^. However, this response and its propagation along the basilar membrane only exists in pristine cochleae, indicating that the photomechanical mechanism is highly dependent on the integrity of the cochlear structure^26,52^. The strongest argument against the direct activation of SGNs through pulsed infrared radiation comes from the findings of two groups who were not able to evoke a response through laser irradiation with infrared light after infusion of neomycin into the scala tympani or after a combined treatment with kanamycin and furosemide. At the same time, the groups were able to evoke a response with monopolar electrical stimulation at the round window of the cochlea^21,22,24,25^. The logical argument was that local heating in the cochlea during INS generates a pressure wave, which vibrates the basilar membrane and stimulates hair cells directly.

Since most induced hearing loss animal models have residual hearing, especially at low frequencies, a photoacoustic effect was thought to be sufficient to explain the auditory responses induced by INS^20,23,25^. However, the arguments in favor of exclusive photoacoustic effects of INS in these deaf animals leave some questions open. Assuming that response to INS only comes from a mechanical stimulation of hair cells, the model would not explain why only localized stimulation at high frequencies is possible in partially deaf animals^53^, why that stimulation only occurs within the beam path of the radiation^10,26,54,55^ and why that laser responses cannot be masked with acoustic stimuli in animals with remaining hearing but elevated thresholds^56,57^.

To address the controversy, an animal model whose cochlea does not possess hair cells but does have SGNs and a functioning nerve is required. Unfortunately, if all hair cells are removed, the neurons typically do not survive and nerve function is absent^58^. In the next paragraph three deaf animal models will be used to tackle the controversy described above.

### INS in deaf animals

INS was previously used to evoke auditory responses oABRs in gerbils, guinea pigs^2,59,60^, mice^14^, and cats^61^. However, no successful stimulation of congenitally deaf animals has thus far been reported. The results of this study demonstrate for the first time that auditory neural responses can be evoked with INS in congenitally deaf animals. Although none of the three congenitally deaf mice strains showed any response to acoustic stimulation, response to INS was recorded in two of the mutant stains. The results also show that the responses induced by INS were highly dependent on the presence of SGNs. SGNs were present in *Vglut3* ^−/−^ mice and at the apex of the *Neurog1* KI, but were almost completely lost in *Atoh1* CKO mice. Sites along the cochlea where INS induced oABRs could be evoked correlated with the location of the SGNs on microCT imaging. The severe loss of SGNs likely makes it impossible to evoke an oABR in *Atoh1* CKO mice.

#### No response in mice lacking hair cells and spiral ganglion neurons

In *Atoh1* CKO mice, the loss of *Atoh1* expression starting at an early stage of development (E18.5) triggers hair cell and supporting cell death. All the IHCs and the majority of OHCs are lost within 3 weeks after birth, and the entire OC appears as a flat epithelium after 3 months^32^. In addition to hair cell loss, the majority of the SGNs are lost at 3–5 months. These animals lack all target structures for acoustical and optical stimulation, namely, the hair cells and spiral ganglion neurons. As expected, no responses to acoustic and optical stimulation were recorded in our experiments.

#### Responses to INS in *Neurog1* KI mice

In *Neurog1* KI mice, the loss of *Atoh1* expression and replacement of *Neurog1* results in development of more hair cells. However, the manipulation alters the gene expression profiles in both hair cells and supporting cells. These alterations lead to only a patchy loss of OHCs in neonatal mice, but apparently lead to a more massive long-term loss of hair cells, supporting cells and SGNs in the middle to basal cochlea in adult animals as shown in our X-ray microCT imaging. The deafness of *Neurog1* KI mice is likely caused by a structurally disorganized cochlea and variably differentiated hair cell stereocilia. The inability to evoke an auditory response with acoustic stimuli up to 107 dB SPL in *Neurog1* KI mice and the ability to evoke responses with the laser supports the view that the stimulation occurs through the direct interaction between the hair cells or SGNs and the neuron. Still, in *Neurog1* KI animals, direct activation of the IHCs by INS is possible and may account for the oABRs as long as the synaptic connections between IHCs and SGNs are intact.

#### *Vglut3*^−/−^ mice, which have no synaptic transmission at the hair cell respond to INS

*Vglut3* ^−/−^ mice have normal hair cell counts and functional SGNs. However, no transmitter release was observed when hair cells were directly depolarized^34–36^. The lack of stimulation is based on a lack of transmitter originating from a loss of vesicle filling. This makes the model ideal to test the contribution of hair cells. No auditory responses were recorded in response to acoustic stimuli but oABRs were evoked by INS. These animals are deaf because the normal path of mechanoelectrical transduction in the cochlea is interrupted due to the inability of the hair cell to stimulate the nerves. However, the neurons are still present and can be stimulated directly, which has been done by direct irradiation with infrared light.

Note that the type II auditory nerve fibers innervating the OHCs are intact in *Vglut3* ^−/−^ mice. One may argue that INS directly activates OHCs. It is possible that type II neuron activity may account for the oABRs induced in these mice. Although further evidence is needed, we assume that laser evoked activity originating from OHCs and type II auditory nerve fibers does not account for the oABR in *Vglut3* ^−/−^ mice. The assumption is based on the following: (1) type II neurons only compose of about 5–10% of the SGNs, so the response would be small to absent. However, the oABRs evoked in *Vglut3* ^−/−^ mice are only slightly smaller to that of the normal hearing animals (Fig. 5). (2) To our knowledge, no ABR or CAP responses dominated by the responses of type II auditory nerve fibers have been reported. (3) Type II nerve fibers are supposed to be activated only at extremely high sound levels while the oABR of *Vglut3* ^−/−^ mice was inducible at low energy levels, similar to those of normal hearing animals.

### Possible mechanisms of INS as a result from spatially and temporally confined heating

The objective of the study was not to investigate the mechanism of INS. Rather, the experiments were designed to identify whether irradiation of only SGNs could result in an auditory response or whether hair cells are required. The experiments did not examine the mechanism for INS, which has been done in several previous studies. In the following section, findings which can explain the mechanism for INS if only SGNs are involved will be discussed.

#### INS leads to rapid, spatially confined temperature changes

During INS, the fluid in the target tissue absorbs the photons and their energy is converted into heat^6–10^. The time over which the energy is delivered is typically between 10 µs and 10 ms. Furthermore, the radiant exposure is below 1 J/cm^2^. The parameters are described as thermally confined, which indicates that the heating of the irradiated volume occurs for a time short enough that the heat delivered to the target volume cannot be disseminated during the same time course. The heating of the target volume has been measured by many groups using a thermal camera^6^ or the heat dependent resistance of pipettes in the beam path^7,8,10^. The temperature changes at a given time point agrees with the theoretical predictions for the heat dissipation in water^10,62^.

#### Heating activates temperature sensitive Ion channels

Spatially and temporally confined heating of a target volume may also activate temperature sensitive ion channels. The candidates include the transient receptor potential (TRP) family^9,14,15,63^. Transient receptor potential channels (vanilloid) (TRPV) are activated by temperature changes and immunohistochemistry has shown that TRPV1 and TRPV4 channels are expressed in SGNs^14,64^. *In vitro* studies have demonstrated that irradiation with a pulsed infrared laser activates the channels at temperatures that have previously been reported as the activation temperature in the literature^9,15^. In an *in vivo* study using a knockout mouse lacking the TRPV1 channel did not show a response to INS^14^. The results have shown that the heat sensitive channels open if their activation temperature is reached. It has been discussed that the channels play a role in the generation of an action potential during INS^6,9,14,15,63^. This is different from the suggestion that GABAergic transmission^16^ is modulated by heating or that channel activation occurs via a second messenger^17^.

#### Heating leads to an increase in cell membrane capacitance

In his classic paper, Parker^12^ describes the effects of temperature jumps on membrane currents in *Xenopus* oocytes. The membrane currents were recorded under voltage clamp. Two components could be identified, a slow maintained current, which inverted its direction at about 25 mV membrane holding potential and a fast transient inward current with the onset of the heating and a fast transient outward current at the end of the heat jump. The direction of the current did not change for holding potentials between −160 and + 30 mV. It has been suggested that sodium ions (Na^+^) and protons (H^+^) carry the slow component and that a displacement of charges across the membrane leads to the transient currents. Similar experiments studying the fast component have been repeated with an infrared laser in *Xenopus* oocytes, Human Embryonic Kidney cells (HEK293), vestibular hair cells, neurons, outer hair cells, spiral ganglion neurons, cardiomyocites, and artificial lipid membranes^7,8,11,13,41,65^. The transient inward current has been found in all of the model systems. During laser irradiation, the resulting rapid temperature change (dT/dt) leads to capacitive changes in the cell membrane. Modeling efforts suggest that the capacitive changes are based on changes in membrane thickness^66^.

#### Heating leads to an increased intracellular calcium concentration

In addition to the rapid changes in temperature, the slow changes (ΔT) should be considered as well. Parker^12^ postulated that leakage currents originate from heating and that the currents are carried by Na^+^ and H^+^ ions. However, his experiments did not support the involvement of calcium ions in the generation of the slow currents. This is in contrast to experimental results showing that the intracellular calcium concentration is elevated during heating^7,8,18,19^ and leads to the contraction of cardiomyocites^18,67^, the activation of calcium dependent potassium channels^8^, or the change of ion channel kinetics^6^.

#### Laser irradiation result in membrane poration

One of the first reports on laser irradiation as a method to stimulate neurons came from Fork’s study on Aplysia^68^. During the laser irradiation, micro holes were created depolarizing the cell. The experiments differ from the more recent efforts in stimulating neurons with infrared (λ = 1440 nm to 2200 nm) radiation. In a recent paper, the authors argue that the change in capacitance, calculated from the membrane current measured under voltage clamp conditions originates from transient, small-diameter nanopores in the membrane^69^.

## Conclusion

The results show that the target structures for INS in deaf animals, the spiral ganglion neurons, must be present and viable to evoke a response. Synaptic transmission between the inner hair cells and the spiral ganglion neuron is not necessary. The exact mechanism by which SGNs are stimulated is still not determined. It appears that many factors must contribute for an action potential to occur. None of the mechanisms alone, described in the paragraph on the mechanism of INS, seem to result in action potential. Rather the combination of several steps is required for a successful stimulation of nerves.

## Methods

### Animals

Three lines of gene manipulated mice of either sex weighing 25–35 g and 3–5 months old were used in this study. The strains included *Atoh1* CKO (N = 6), *Neurog1* KI (N = 6) and *Vglut3* ^−/−^ (n = 12) mice. All strains have the C57BL/6 J background. Normal hearing wild type C57BL/6 J mice (n = 6) were used as controls. In addition, the cochleae of P7 animals of *Atoh1* CKO, *Neurog1* KI and control mice (n = 3 for each strain) were used for immunostaining to verify the presence of nerve fibers. All procedures were carried out in accordance with the NIH Guide for the Care and Use of Laboratory Animals and were approved by the Institutional Animal Care and Use Committee at Northwestern University.

### Surgical access of the cochlea

Animals were anesthetized with an initial intraperitoneal (i.p.) injection of a mixture of ketamine (100 mg/kg) and xylazine (2 mg/kg) diluted 1:20 in Ringer’s Lactated Solution. The maintenance dose of ketamine (20–33% of the original dose) was injected if the animal showed a positive paw withdrawal reflex. The reflex and vital signs including body temperature, O_2_ saturation, respiratory and pulse rate were checked and recorded every 15 minutes. The body temperature of the animals was maintained at 37 °C. After anesthesia was established and cochlear function was determined (see also below) the cochlea was surgically accessed as described previously^14^. In short, the frontal skull was exposed and three screws were placed to mount the head of the animal to a rigid stereotactic head holder (Stoelting, Kiel, WI) using dental acrylic (Methyl methacrylate, Co-oral-ite Dental MFG Co., CA). A C-shaped incision was made behind the left or right pinna. The outer ear canal was exposed by blunt dissection and cut close to the bony skull. The bulla was then exposed and an approximately 3.0 × 0.8 mm^2^ opening was created such that the entire cochlea from the round window to the apex could be seen.

### Acoustic measurements

#### Distortion Product Otoacoustic Emission (DPOAE) Measurements

The voltage commands to drive the speakers in the ER10C system (Ethymoic Research Inc., Elk Grove Village IL) were generated with custom written software in in Testpoint^®^ (SuperLogics, Inc. Massachusetts). Stimuli consisted of 10 ms tone bursts, including a 1 ms rise and fall time. Two acoustic stimuli were presented simultaneously via the two speakers of the ER10C system. The frequency of f_2_ was changed from 25 to 2.5 kHz at 5 steps per octave and the corresponding frequency f_1_ was calculated as the ratio f_2_/f_1_. The ratio was between 1.2 and 1.25. The sampling rate for the D/A board (KPCI 3110, Keithley, Cleveland, OH) was 100 kHz. The level for f_1_ and f_2_ was about 85 dB SPL and was kept constant. The two sound sources were calibrated with a 1/8-inch microphone (Brüel & Kjær North America Inc., Norcross, GA) on a dried mouse half-head coupler.

The resulting DPOAEs were measured with the ER-10C Lo Noise™ DPOAE Probe System. The sensitive calibrated microphone was placed in the outer ear canal and secured with a self-sealing ER10C-02 eartip tube. The voltage of the microphone was recorded at a sampling rate of 100 kHz and the average response to 512 stimulus presentation was stored on the computer. For all experiments the amplifier gain of the ER-10C was set at 0 dB. To analyze the data, a Fast Fourier Transformation (FFT) was performed in MATLAB (MathWorks, Natick, MA) off-line and the frequency spectrum was plotted. The amplitude of the distortion products f_2_-f_1_ and 2f_1_-f_2_ was determined from the resulting spectrum.

#### Auditory Brainstem Response (ABR) measurements

Acoustic stimuli to assess cochlear function through ABR measurements were 12 ms long tone bursts (including a 1 ms rise/fall time) or 50 µs acoustic clicks. The frequency for the tone bursts varied from 32 kHz to 2 kHz, and was varied with 2 steps per octave. The level for both stimuli, tone bursts and clicks, was decreased by steps of 5 dB starting at the maximum level of the speaker, ≤ 107 dB SPL (SPL: sound pressure level re 20 µPa) until a visible response to the acoustic stimuli disappeared. The voltage commands for the acoustic stimuli were generated with custom written software in Testpoint^®^ and were used to drive a Beyer DT 770Pro headphone. A speculum was extended from the speaker and placed directly in the ear canal of the animal. The calibration of the acoustic output was performed with the 1/8-inch Brüel & Kjær microphone.

For ABR measurements to acoustic stimuli, three hypodermic needles were placed at the bulla, vertex and body of the animals, respectively. The electrodes were connected to an ISO-80 differential amplifier, of which the amplification was set at 10,000. The high- and low-pass of the amplifier was set at 300 Hz and 3 kHz, respectively. Responses to acoustic clicks and to tone pips were both measured. The threshold for acoustic ABR or aABR was defined as the sound level required for a visible response in the recorded traces. All the signals were averaged by 50–256 repeated responses, with the sampling rate set at 250 kHz.

During data acquisition, the stimuli and the corresponding responses were visually monitored with an oscilloscope. A confounding factor introducing noise into the measurements is the electrocardiogram (ECG), which is usually at least three times larger in amplitude (~25 µV) than the ABR. The program automatically rejected recordings that contained the ECG by rejecting traces with peak-to-peak amplitudes larger than 15 µV.

### Optical stimulation

Optical pulses (λ = 1860 nm; 100–500 µs in duration) were generated by an infrared laser (Lockheed Martin Aculight Corp., Bothell, WA) at a rate of 5–10 pulses per second (pps). The laser output was coupled to a 200 µm optical fiber (P200-5-VIS-NIR, Ocean Optics, Dunedin, FL) with a numerical aperture of 0.22 in air to deliver the stimuli. The radiant energy was measured with a J50LP-1A energy sensor (Coherent, Santa Clara, California) at the tip of the optical fiber in air and was 164 µJ/pulse for a 100 µs pulse length and at the maximum radiant energy output of the laser.

Tissue between the light delivery system, here the tip of the optical fiber, and the target structure (spiral ganglion neurons) can scatter and absorb the photons and reduce the radiation at the target. To estimate the radiant energy at the target we considered the thickness of the cochlear wall, which was determined from histological sections to be about ~25 µm. For the selected wavelength of the radiation the extinction was small, about 3.5% (unpublished data). Furthermore, the distance between the surface of the cochlear wall and the spiral ganglia was estimated to be ~300 µm. The penetration depth of the radiation at 1860 nm in water is about 771 µm (e.g.^70^). Since the incident energy decreases in water by 1/e for each 771 µm travel distance along the optical path, it was estimated that the energy at the SGN was about 40% of the energy measured at the tip of the optical fiber.

The 200 µm optical fiber was mounted on a micromanipulator (MHW103, Narishige, Tokyo, Japan) and was inserted in close vicinity of the cochlear wall to deliver optical stimulations. Auditory brainstem responses to optical stimulation (oABRs) were then recorded. During the measurements the placement of the optical fiber was moved from the cochlear base (close to the round window) to the cochlear apex until an oABR was detected and the response was maximum (Fig. 3).

### Synchrotron X-ray micro-computed tomography (microCT) Imaging

After the data have been acquired, the animals were euthanized with an overdose of sodium pentobarbital (Euthasol®) and the bullae were harvested and fixed in 4% paraformaldehyde for 12 h. Next, the specimens were decalcified in 10% EDTA for about 2–3 days. MicroCT imaging was carried out using monochromatic radiation with photon energies of 22 kilo-electron volts (keV) at beamline 2-BM of the Advanced Photon Source (APS), Argonne National Laboratory as described before^26,46^. To acquire phase contrast images, the distance between the detector and the tomography rotation axis was set at 600 mm. A 5× objective lens was used in the detector system. The resulting field of view was about 3 × 3 mm^2^. Specimens were placed such that they were in the visual field at all times. A series X-ray projections were taken over a range of 180 degrees at increments of 0.12 degrees, with an exposure time for each frame of 0.2–0.3 s. Flat field images (no object in the beam path) were recorded before and after each image series. A dark field image (the X-ray beam was blocked) was also captured at the end of the scanning. The reconstruction of the cochlea was on a 2048 × 2048 grid using a custom-written phase retrieval software^71^. The reconstruction resulted in a 1.45 µm isotropic voxels. The spatial resolution was determined from the response of the system to a sharp discontinuity in the image such as a bony edge. The gray values along a line crossing a sharp edge was used for the measurements. The parameter measured was the distance required for the gray values to fall from 90% to 10%. The resulting distance was 4.4 µm, which was considered the spatial resolution. From the 3D set of reconstructed images, the presence of the HCs and the SGNs was examined at different locations along the cochlea.

### Immuno-Fluorescence histochemistry of cochlear whole-mount

Immuno-fluorescence histochemistry of cochlear whole-mounts was described in previous publications. Briefly, the cochleae were first fixed by cardiac perfusion with 4% PFA, the ears were dissected, decalcified, prepared and dehydrated in 70% ethanol. After rehydration with graded ethanol and PBS, the whole-mount cochleae were blocked with 2.5% normal goat serum in PBS containing 0.25% Triton X-100 for 1 hour. A combination of the primary antibodies for Myo7a (1:200; Proteus Biosciences, 25-6790) and Neurofilament (1:1000; Millipore, AB5539) were added and incubated for 48 hours at 4 °C. After several washes with PBS, corresponding secondary antibodies (1:500; Molecular Probes Alexa fluor 647, 532, or 488; Invitrogen) were added and incubated overnight at 4 °C. The cochleae were washed with PBS and mounted in glycerol and images were taken with a Leica TCS SP5 confocal microscope using appropriate excitation/emission settings.

### Data Analysis

The FFT of the original waveforms of DPOAEs and the measurement of the cubic (2f_1_-f_2_) and quadratic (f_2_-f_1_) distortion products were performed in MATLAB. The results were plotted using IGOR Pro (Wavemetrics, Lake Osweqo, OR). Averages and standard deviations were calculated for the ABR thresholds and DPOAE amplitudes obtained for different frequencies. An analysis of variance (ANOVA) was performed. If the ANOVA indicated differences among the means, a posteriori test was used for making pairwise comparisons among the means. The honestly significant difference (HSD) test by Tukey was used. The tests are part of a statistical package provided by IGOR Pro. Statistical decisions were made for a probability of 0.05.
